# Mass Spectrometry Imaging Differentiates Chromophobe Renal Cell Carcinoma and Renal Oncocytoma with High Accuracy

**DOI:** 10.7150/jca.47698

**Published:** 2020-08-21

**Authors:** Mark Kriegsmann, Rita Casadonte, Nadine Maurer, Christine Stoehr, Franziska Erlmeier, Holger Moch, Kerstin Junker, Christiane Zgorzelski, Wilko Weichert, Kristina Schwamborn, Sören-Oliver Deininger, Matthias Gaida, Gunhild Mechtersheimer, Albrecht Stenzinger, Peter Schirmacher, Arndt Hartmann, Joerg Kriegsmann, Katharina Kriegsmann

**Affiliations:** 1 Institute of Pathology, Heidelberg University, Heidelberg, Germany.; 2 Proteopath Trier, Trier, Germany.; 3 Institute of Pathology, University Hospital Erlangen-Nürnberg, Erlangen, Germany.; 4 Institute of Pathology and Molecular Pathology, University Hospital Zurich, Switzerland.; 5 Department of Urology and Pediatric Urology, University of Saarland, Homburg/Saar, Germany.; 6 Institute of Pathology, TU Munich, Munich, Germany.; 7 Bruker Daltonik, Bremen, Germany.; 8 Institute of Pathology, University of Mainz, Germany.; 9 Centre for Histology, Cytology and molecular Diagnostics Trier, Trier, Germany.; 10 Danube Private University, Krems, Austria.; 11 Department Hematology, Oncology and Rheumatology, Heidelberg University, Heidelberg, Germany.

**Keywords:** Oncocytic renal tumors, chromophobe renal cell carcinoma, renal oncocytoma, mass spectrometry imaging, proteomics

## Abstract

**Background:** While subtyping of the majority of malignant chromophobe renal cell carcinoma (cRCC) and benign renal oncocytoma (rO) is possible on morphology alone, additional histochemical, immunohistochemical or molecular investigations are required in a subset of cases. As currently used histochemical and immunohistological stains as well as genetic aberrations show considerable overlap in both tumors, additional techniques are required for differential diagnostics. Mass spectrometry imaging (MSI) combining the detection of multiple peptides with information about their localization in tissue may be a suitable technology to overcome this diagnostic challenge.

**Patients and Methods:** Formalin-fixed paraffin embedded (FFPE) tissue specimens from cRCC (n=71) and rO (n=64) were analyzed by MSI. Data were classified by linear discriminant analysis (LDA), classification and regression trees (CART), k-nearest neighbors (KNN), support vector machine (SVM), and random forest (RF) algorithm with internal cross validation and visualized by t-distributed stochastic neighbor embedding (t-SNE). Most important variables for classification were identified and the classification algorithm was optimized.

**Results:** Applying different machine learning algorithms on all m/z peaks, classification accuracy between cRCC and rO was 85%, 82%, 84%, 77% and 64% for RF, SVM, KNN, CART and LDA. Under the assumption that a reduction of m/z peaks would lead to improved classification accuracy, m/z peaks were ranked based on their variable importance. Reduction to six most important m/z peaks resulted in improved accuracy of 89%, 85%, 85% and 85% for RF, SVM, KNN, and LDA and remained at the level of 77% for CART. t-SNE showed clear separation of cRCC and rO after algorithm improvement.

**Conclusion:** In summary, we acquired MSI data on FFPE tissue specimens of cRCC and rO, performed classification and detected most relevant biomarkers for the differential diagnosis of both diseases. MSI data might be a useful adjunct method in the differential diagnosis of cRCC and rO.

## Introduction

Classification of primary renal tumors is a common task in routine histopathological diagnostics. While the differentiation of most renal tumors is possible on morphology alone, some require additional histochemical, immunohistochemical (IHC) or molecular investigations. A frequent scenario where additional studies are recommended is in the differential diagnosis of oncocytic renal tumors 1. While several renal tumors exhibit a certain degree of cytoplasmic eosinophilia, the differential diagnosis of chromophobe renal cell carcinoma (cRCC), eosinophilic variant (which comprise about 40% of all chromophobe renal cell carcinomas) and renal oncocytoma (rO) is particularly challenging 2. Although typical morphological, histochemical and genetic characteristics have been described for both tumors, a subset of cases remains difficult to classify 3. However, correct classification of cRCC and rO is of utmost importance as the former, although associated with a good prognosis, has the potential to progress and metastasize 4.

While tumor cells in both tumors generally show a nested or broad trabecular unencapsulated growth and eosinophilic finely granular cytoplasm, typical morphological features of cRCC are distinct cell borders, raisinoid, irregular, wrinkled and angulated nuclei, bi- or multinucleation, perinuclear halos and fibrovascular stroma. Typical morphological characteristics of rO include more closely arranged tumor cells at the periphery of the lesion, presence of oncoblasts, uniform small, round centrally located nuclei, and myxoid or hyalinized stroma (central scar). One should be aware that rO may exhibit perinephric or renal sinus fat invasion, venous invasion, degenerative atypia with bizarre pleomorphic nuclei and mitosis 5-8.

A helpful histochemical stain in the differential diagnosis is the colloidal iron stain (Hale stain). While cRCC frequently exhibit intense reticular staining, rO show less intense fine dust-like reactivity 9. However, the stain is difficult to standardize and utility in the diagnosis of the eosinophilic variant of cRCC has been doubted 1.

**Figure 1:**
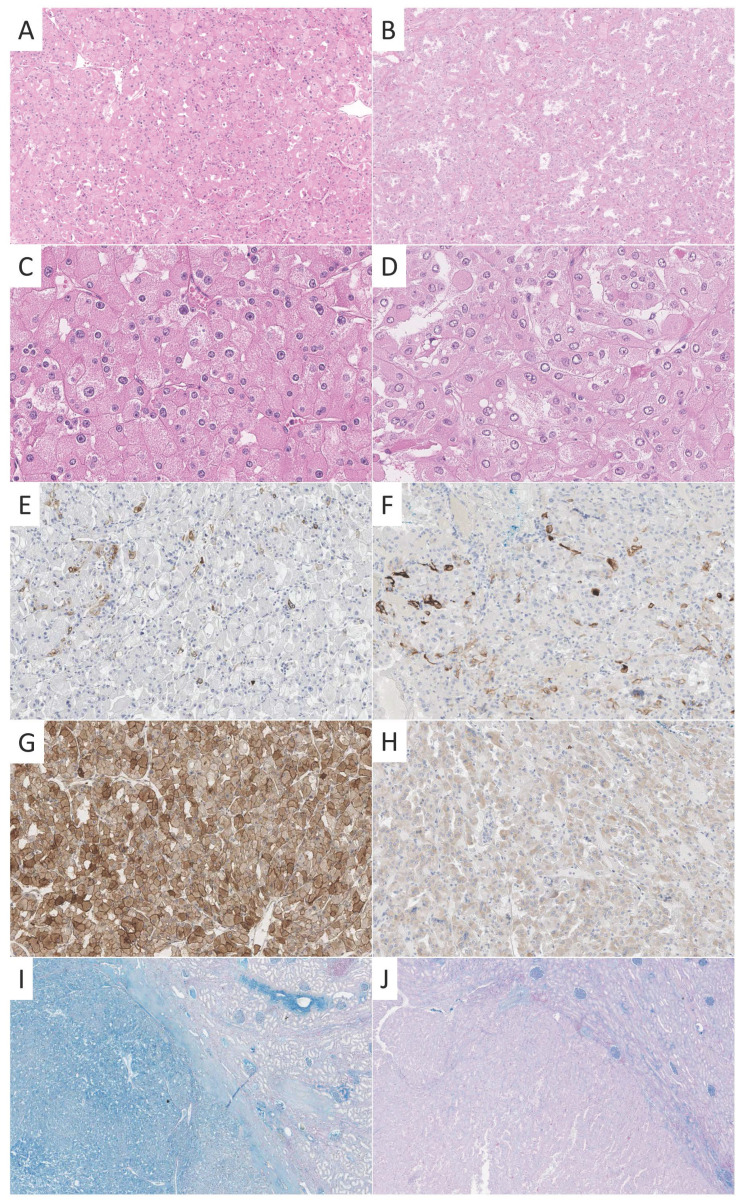
** Examples of different stainings of cRCC and rO.** One exemplary case of cRCC (A, C, E, G, I) and rO (B, D, F, H, J) is shown. On the HE stained tissue sections typical morphological features can be appreciated (A, B, 100x; C, D, 400x). CK7 is positive in scattered cells in both cases (E, F, 100x). CD117 shows strong membranous and cytoplasmic staining in cRCC and weak cytoplasmic reactivity in rO (G, H, 100x). This staining pattern is not specific as both tumors may show strong or weak reactivity. HALE colloidal iron stain shows positivity in cRCC and negativity in rO (left) which can be best appreciated in comparison to surrounding renal parenchyma (right, I, J, 20x).

A survey among urologic pathologists reported cytokeratin (CK) 7 (94%) as most frequently used and most helpful adjunct IHC stain in the differential diagnosis of cRCC and rO 3. In this study, a positivity of CK7 in less than 5% of tumor cells was regarded as most supportive of rO and diffuse uniform staining as confirmatory for cRCC. There was less agreement weather negative or focal staining of CK7 was compatible with cRCC but is a feature that can be observed according to our experience (**Figure 1**).

With regard to genetic changes, losses of multiple chromosomes by karyotyping was regarded supportive of cRCC by most (65%), and chromosomal gains only by some (18%) in the same study 3. However, rO might often show a diploid karyotype and also chromosomal losses 10.

In an attempt to better characterize the proteomic landscape of renal tumors, mass spectrometric methods have been applied 11. Among those, mass spectrometry imaging (MSI) has prompted interest among pathologists, as it combines the detection of multiple proteins or peptides with information about their topographical localization within tissue sections. The method was previously used to classify different cancer subtypes 12-14. However, this technique has rarely been applied to study renal tumors and data on the differential expression patterns in cRCC and rO is lacking so far 15-17. Additionally, it is not yet clear how to standardize the analysis of the complex data generated. To improve diagnostic accuracy we analyzed a total of 135 oncocytic tumors including 71 cRCC and 64 rO by MSI, developed a mathematical model and evaluated the most important peptides to differentiate both tumor types from one another.

## Patients and Methods

### Patients and data collection

The cohort consisted of formalin-fixed paraffin embedded (FFPE) specimens from patients with diagnosed with either cRCC (n=71) or with rO (n=64). Diagnosis was made according to the recommendations of the 2016 World Health Organization classification of renal 10. Where appropriate, additional histochemical and immunohistochemical stainings were performed (**Figure 1**). Tissue blocks were extracted from the archive of the Institute of Pathology Erlangen, with the support of the local tissue bank according to standards defined by the local ethics committee (Erlangen, vote from 18.01.2005; #3755; 12.02.2008; #329_16B and 14.11.2016; #3755). Tissue microarray (TMA) construction was performed as previously described 18. In short, one representative punch from each tumor was transferred to a new block for TMA construction 19. Representative cases of cRCC and rO were stained by CK7 and CD117 and HALE for illustrative purposes (for staining properties see **Supplementary Table 1**).

### Proteomic characterization by matrix assisted laser desorption/ionization mass spectrometry imaging

Three micrometer thick sections of the TMA were cut and mounted onto conductive indium tin oxide-coated glass slides (Bruker Daltonik, Bremen, Germany). Sample slides were processed for dewaxing with xylene (Fischer Scientific, Schwerte, Germany), rehydrated through graded ethanol washes (Fischer Scientific), and subjected to heat induced antigen retrieval in 10 mM tris buffer at pH 9.0 at 95 °C for 20 min, as previously described 14. For on-tissue digestion, trypsin solution was prepared in 200 µl 40 mM ammonium bicarbonate (Sigma-Aldrich, Taufkirchen, Germany) to a final concentration of 0.1 µg/µl and sprayed with an automatic reagent sprayer (ImagePrep, Bruker Daltonik) in 25 cycles with a fixed nebulization time of 1.2 s. Sections were subsequently incubated in a humidity chamber at 37 °C for 1.5 h. A solution of 7 mg/ml of alpha-cyano-4-hydroxycinnamic acid matrix (Bruker Daltonik) in 50% acetonitrile/0.5% trifluoroacetic acid (Fischer Scientific) was then applied onto digested sections using the same matrix spraying device with an optimized Bruker Daltonik default method for sensor controlled nebulization of the matrix.

MSI was performed using an Autoflex Speed matrix-assisted laser desorption/ionization time-of-flight (TOF)/TOF mass spectrometer (Bruker Daltonik) operated in reflectron mode with positive polarity and equipped with a smartbeam laser. MSI runs were programmed using flexControl and flexImaging software (Bruker Daltonik). Each spectrum was automatically generated at a spatial resolution of 150 µm (Bruker Daltonik) in the mass range of m/z 500-5000. 1600 laser shots were acquired for each spectrum with 2 kHz repetition rates. A peptide calibration standard mix including bradykinin, angiotensin II, angiotensin I, substance P, bombesin, ACTH clip 1-17, ACTH clip 18-39, and somatostatin 28 (Bruker Daltonik) was used for external calibration. Following MSI measurements, matrix was removed by two washes in 100% methanol (Fischer Scientific) for 5 min each followed by hematoxylin and eosin (HE) staining.

### Tumor annotation, data processing, and extraction

Tissue sections analyzed by MSI and stained by HE were scanned by a slide scanner (Aperio AT2, Leica Biosystems, Wetzlar, Germany) and the tumor regions were annotated using SCiLS Cloud (SciLS GmbH, Bremen, Germany). MSI data were processed using SCiLS Lab (SCiLS GmbH) for peak- and image visualization. Annotations were imported into SCiLS Lab software and spectra were corrected to baseline using the algorithm TopHat and normalized by total ion count. After manual peak picking, the mean intensity of reprocessed spectral peaks of each patient was exported for further statistical analysis.

### Statistical analyses

All statistical analyses were performed in R-Statistical Software (version 3.4.3, Free Software Foundation) and R-Studio (version 1.1.383, Affero General Public License, Boston, USA).

*t*-distributed stochastic neighbor embedding (t-SNE) was performed to visualize the separation of cRCC and rO samples based on the high-dimensional m/z peak intensities data set (package Rtsne, v. 0.15)^20^. In order to visualize similarity between m/z values and/or patients, unsupervised hierarchical K-means (n=2) clustering was performed (distance method: euclidean distance, linkage method: Ward's minimum variance) in R-Statistical software (ComplexHeatmap package, v. 2.4.2).

Different linear, nonlinear and more advanced machine learning algorithms (linear discriminant analysis [LDA], classification and regression trees [CART], k-nearest neighbors [kNN], support vector machines [SVM], and random forest [RF]) with internal cross validation were applied to calculate the accuracy and variable importance of tissue type prediction based on all and selected m/z values (package caret, version 6.0-81)^21^. Graphics were arranged in Inkscape (version 0.92.2).

## Results

### Proteomic MSI analysis can be successfully performed on cRCC and rO FFPE samples

**Figure 2:**
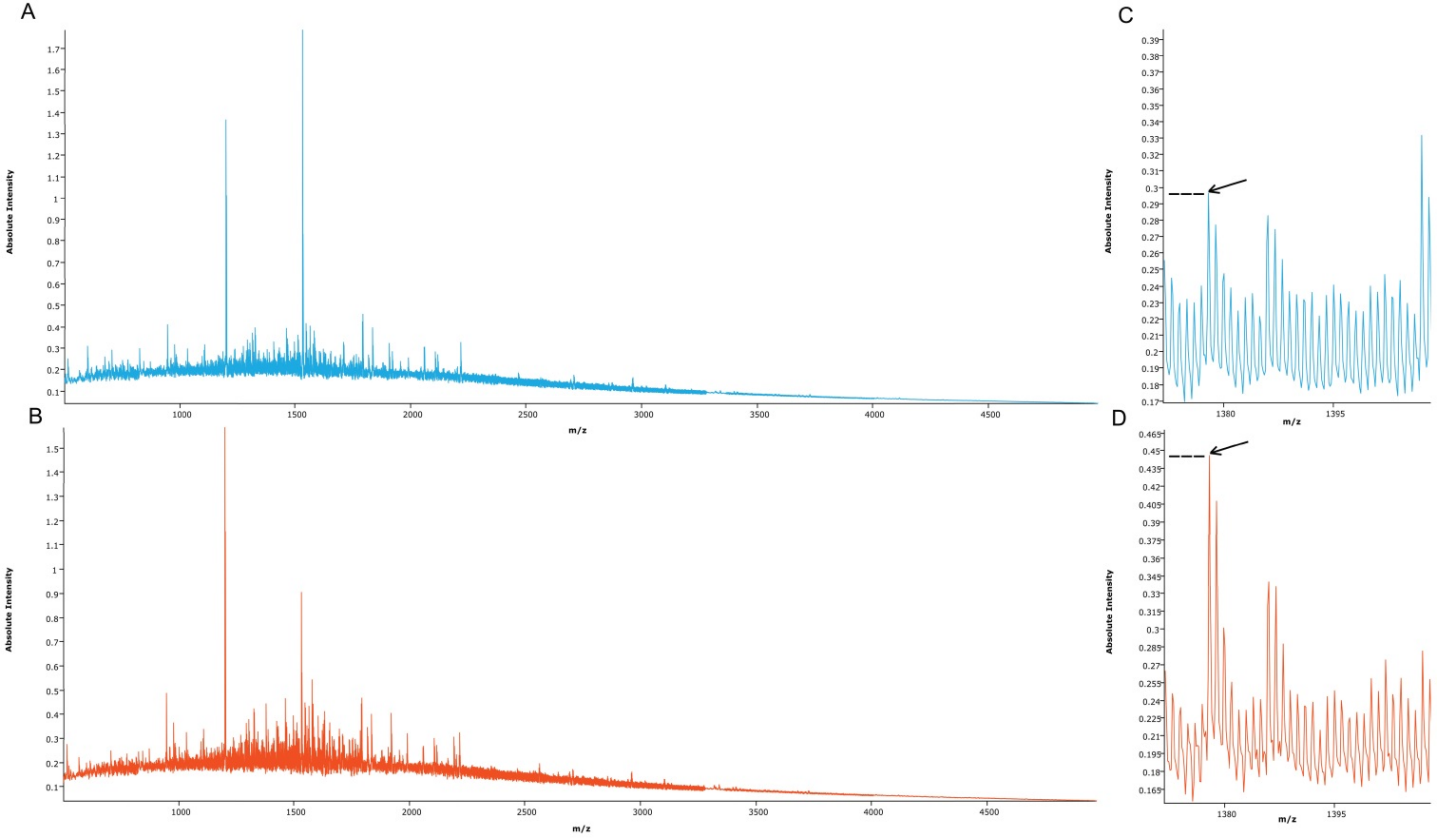
** Mean spectra obtained from cRCC and rO samples.** Mean spectra from all cRCC (A, light blue, n=71) and all rO (B, red, n=64) as well as a peptide peak at m/z 1377.6 (C, D, arrow) are displayed. Discriminant absolute intensities of this peak can be observed.

Proteomic MSI analysis was successfully performed on cRCC (n=71) and rO (n=64) FFPE tissues. 159 peptide peaks in the m/z range from 515.1 and 4953.4 were detected and selected for further statistical analysis. The mean MSI peak intensity of all peaks ranged between 0.05 and 1.59 regardless of tissue type. **Figure 2** shows the sum spectra obtained in the MSI analysis.

### Proteomic MSI allows correct classification of cRCC and rO tissue specimens

Aiming future classification of unknown tissue samples a t-SNE analysis and five different machine classification algorithms have been applied. An internal cross validation approach was performed to calculate the prediction accuracy of each machine classification algorithm.

**Figure 3:**
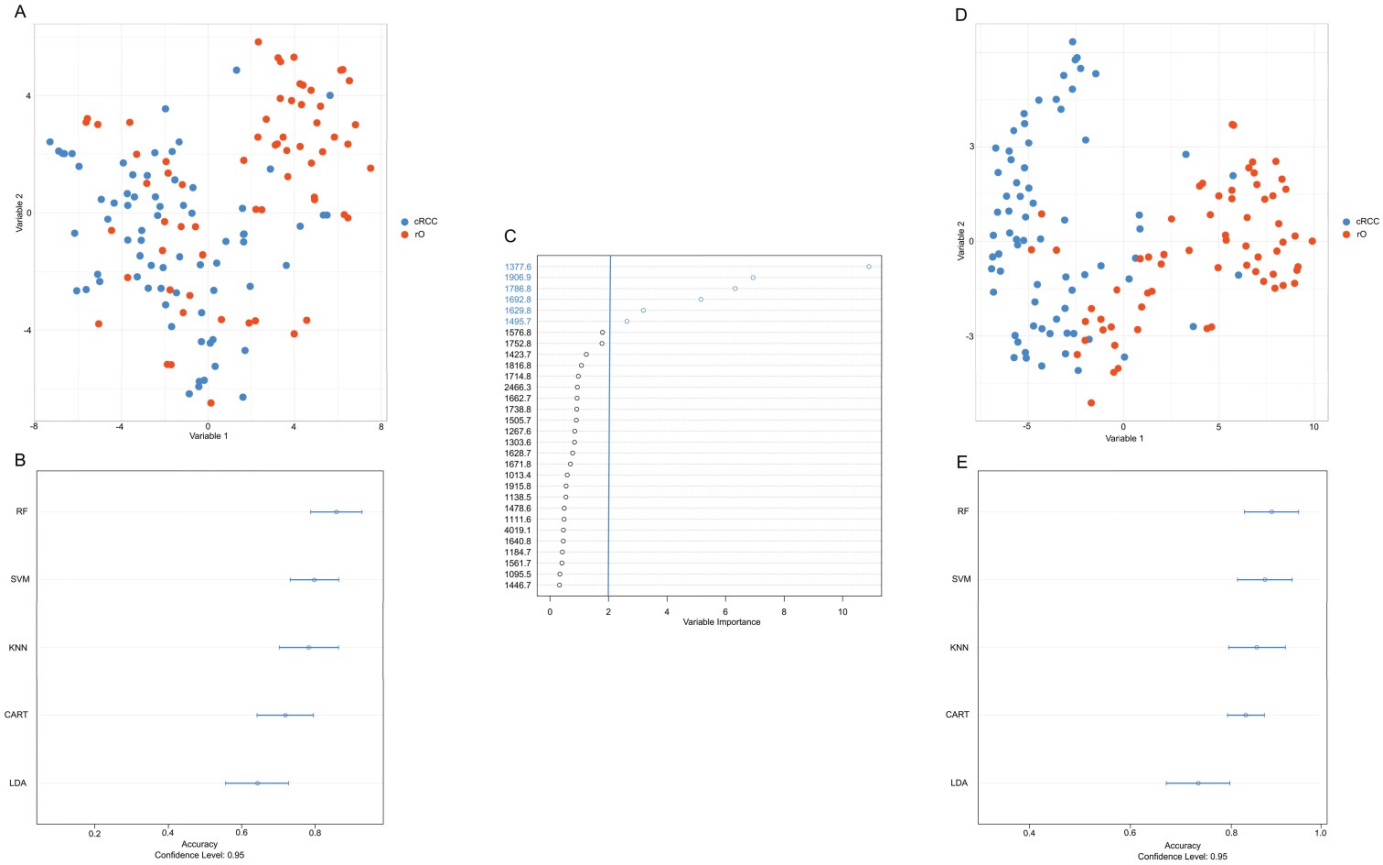
** Development of an accurate classification algorithm.** To reduce the high-dimensional dataset obtained from peptide MSI (intensities of 159 m/z peaks per sample) and to visualize the results a t-distributed stochastic neighbor embedding (t-SNE) analysis was performed (A, D). Common machine classification algorithms were chosen in order to perform classification between cRCC and rO samples based on m/z intensities obtained from MSI (B, E). A cross validation method was chosen to determine the classification accuracy.m/z peaks were ranked by their importance to contribute to a correct classification of cRCC and rO (C). Overall, the figure shows that reduction of variables (i.e. m/z peaks) to six most important improved the classification accuracy. This is reflected in a clearer distinction of cRCC and rO by t-SNE (D) and higher classification accuracy by different machine classification algorithms (E) after variable reduction. Abbreviations: CART, classification and regression tree; KNN, k-nearest neighbors; LDA, linear discriminant analysis; RF, random forest; SVM, support vector machine.

The first differentiation approach including all available m/z peaks (n=159) resulted in low visual separation accuracy with t-SNE (**Figure 3A**). Similarly, applying different machine learning algorithms under consideration of all m/z peaks classification accuracy between cRCC and rO of 85%, 82%, 84%, 77%, and 64% was reached for RF, SVM, KNN, CART and LDA (**Figure 3B**).

Under the assumption that a reduction of variables (i.e. m/z peaks) would lead to a higher classification accuracy m/z peaks were ranked based on their variable importance contributing most to the differentiation between cRCC and rO in the RF algorithm. The six most important m/z values for cRCC and rO classification in descending order starting from the most important were: m/z 1377.6, m/z 1906.9, m/z 1786.8, m/z 1692.8, m/z 1629.8 and m/z 1495.7 (**Figure 3C**). Representative mass spectrometry images of cRCC and rO are displayed in **Supplementary Figure 1.**

Reduction of variables (i.e. m/z peaks) to six most important resulted in an improvement of visual separation between cRCC and rO in t-SNE (**Figure 3D**), which can also be seen in unsupervised cluster analysis (**Supplementary Figure 2**). Moreover, the median classification accuracy of the machine learning algorithms improved to 89%, 85%, 85%, and 85% for RF, SVM, KNN, and LDA and remained at the level of 77% in CART algorithm (**Figure 3E**). Having the highest classification accuracy RF high be the most suitable classification algorithm between cRCC and rO based on proteomic MSI data. The sensitivity and specificity of the final tuned RF model was 89% and 81% respectively.

### Compared to cRCC rO shows higher intensity of all but one m/z peaks contributing to classification

**Figure 4:**
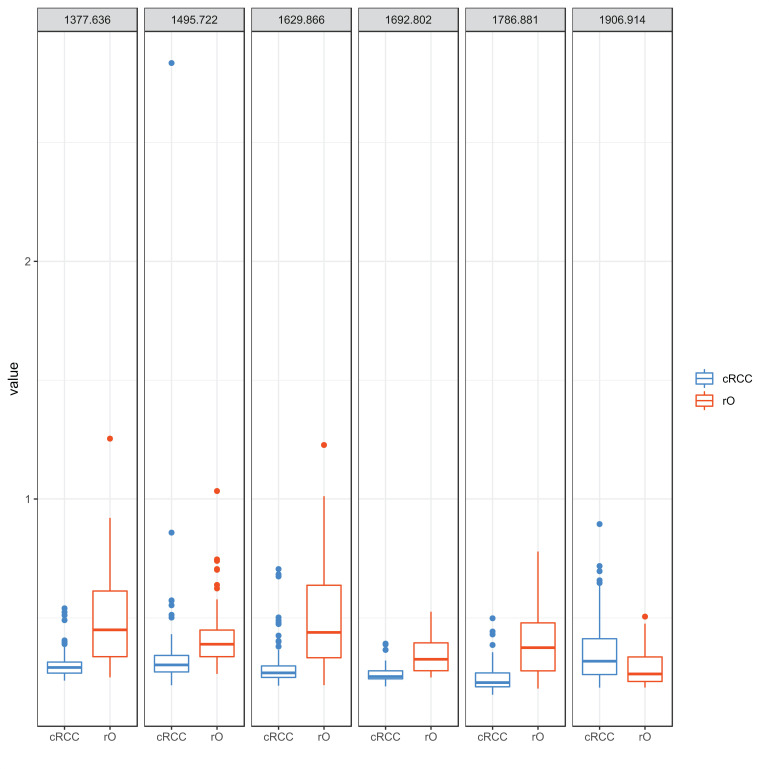
** Peptide intensity profile of selected m/z peaks in cRCC and rO.** The figure visualizes the intensity of selected m/z peaks in the 71 cRCC and 64 rO as boxplots. The six m/z peaks obtained from proteomic analysis that contributed most to the differentiation between cRCC and rO are shown. Each of the outlier dots refers to one patient sample.

The peaks m/z 1377.6, m/z 1906.9, m/z 1786.8, m/z 1692.8, m/z 1629.8 and m/z 1495.7 were identified as most important peaks in contributing to differentiation between cRCC and rO by RF. All but m/z peak 1906.9 (cRCC 0.32 [range 0.21-0.90], rO 0.26 [0.21-0.51]) showed a higher median peak intensity in rO compared to cRCC: m/z 1377.6 - cRCC 0.29 (range 0.24-0.54), rO 0.45 (0.25-1.25); m/z 1786.8 - cRCC 0.23 (range 0.18-0.50); rO 0.37 (0.20-0.78); m/z 1692.8 - cRCC 0.25 (range 0.21-0.29); rO 0.33 (0.25-0.53); m/z 1629.8 - cRCC 0.27 (range 0.21-0.71); rO 0.44 (0.22-1.23) and m/z 1495.7 - cRCC 0.30 (range 0.22-2.83); rO 0.39 (0.26-1.03), (**Figure 4**).

## Discussion

Reliable subtyping of oncocytic renal cell neoplasms into cRCC and rO is important for patient management. Correct classification is often possible by thorough morphological investigation alone, but requires additional histochemical, IHC and genetic studies in some cases. Even using an extended diagnostic arsenal, some tumors remain difficult to classify.

Peptide MSI on FFPE tissue has been suggested to aid classification of tumors as multiple peptides can be detected on a single tissue section, and because the analysis is reliable, tissue saving, rapid and cost-effective 22-25. MSI has previously been used to successfully study non-tumor kidney diseases 26-29 and renal cancer 15-17. However, in the latter investigations mainly papillary and clear cell renal cell carcinoma have been analyzed and data on cRCC and rO provided in the current study were lacking so far. Particularly, peptides that differ between both entities and that can be detected by MSI have not been identified.

Moreover, we introduce a novel workflow of how the classification of different tumors may be improved by using a RF model for classification, variable selection and classification performance improvement. RF classification has previously been applied for classification of peptide MSI data, but not for improving MSI classification models 30-32. Tree-based variable selection methods such as the RF method applied in our study tend to perform better on large datasets such as our dataset compared to classic regression-based models 33. Additionally, we show how the dimensions of the data can be reduced by t-SNE in order to allow visualization of the results which is regarded as the current state-of-the art method for nonlinear dimensionality reduction and visualization 34. This approach has not been applied for peptide MSI classification visualization to the best of our knowledge.

The combination of both mathematical methods, RF classification and t-SNE, allowed to identify m/z 1377.6, m/z 1906.9, m/z 1786.8, m/z 1692.8, m/z 1629.8 and m/z 1495.7 as most important markers for the differential classification of cRCC and rO. The overall accuracy of MSI for the classification of both diseases was 89%. Due to the difficulties to establish a reliable colloidal iron stain and the demanding evaluation, it is difficult to compare the accuracy of MSI data and the colloidal iron stain in both tumors. Likewise, data on the expression of CK7 in cRCC and rO is challenging to compare to MSI data, as reliable cut-off values to define positivity are not firmly established. Today, it is clear that the amount and staining pattern of CK7 positive cells is important for the interpretation of CK7. Some authors even suggested to designate CK7 as negative, even when up to 10% of cells express weakly CK7 35. Other authors state that < 5% CK7 expression is most suggestive of oncocytoma while a negative CK7 staining result is less uniformly considered supportive 3. While earlier investigations reported CK7 positivity in only 27% of oncocytomas 36, latter investigations found CK7 expression in 100% 37. As up to 100% of cRCC are also positive 38 and some low-grade oncocytic tumors show an untypical staining pattern 39, MSI data might be a useful adjunct in the differential diagnosis of cRCC and rO.

The second most common immunohistochemical marker for the differential diagnosis of oncocytic renal cell neoplasms is CD117. However, CD117 does not discriminate between cRCC and rO as about 85% are positive 40, but this marker is useful to discriminate cRCC and rO from oncocytic papillary renal cell carcinoma, oncocytic angiomyolipoma and others 1.

Another diagnostic and rather clinical relevant challenge is the identification of hybrid oncocytic tumors with an admixture of areas (or cells) typical of cRCC and rO 41. Imaging of peptide differences on tissue sections by MSI might facilitate the identification of these areas. However, this has to be investigated on a suitable dataset as these tumors were not included in our study.

Of note, the MSI data were acquired on a mass spectrometer of the former generation. In our experience, even more peptide peaks can be detected on the newer compared to the older instrumentation; however it is not clear whether classification results might be improved by analysis on the most recent generation of mass spectrometers 42. Additionally, the results have been generated on highly standardized TMAs. It is not clear if the results can be simply transferred to whole sections. In this regard, we think that future MSI investigations on cRCC and rO are necessary to draw final conclusions.

In summary, we acquired MSI data on FFPE tissue specimens of cRCC and rO, performed classification and detected most relevant biomarkers for the differential diagnosis of both diseases.

## Supplementary Material

Supplementary figures and tables.
