# Supplementary material for: Mass Spectrometry Imaging Differentiates Chromophobe Renal Cell Carcinoma and Renal Oncocytoma with High Accuracy
Source: J Cancer. 2020 Aug 21;11(20):6081–9. doi: 10.7150/jca.47698 (PMC7477404; doi:10.7150/jca.47698)
Supplement: Supplementary file 1 — Supplementary figures and tables. [file jcav11p6081s1.pdf]

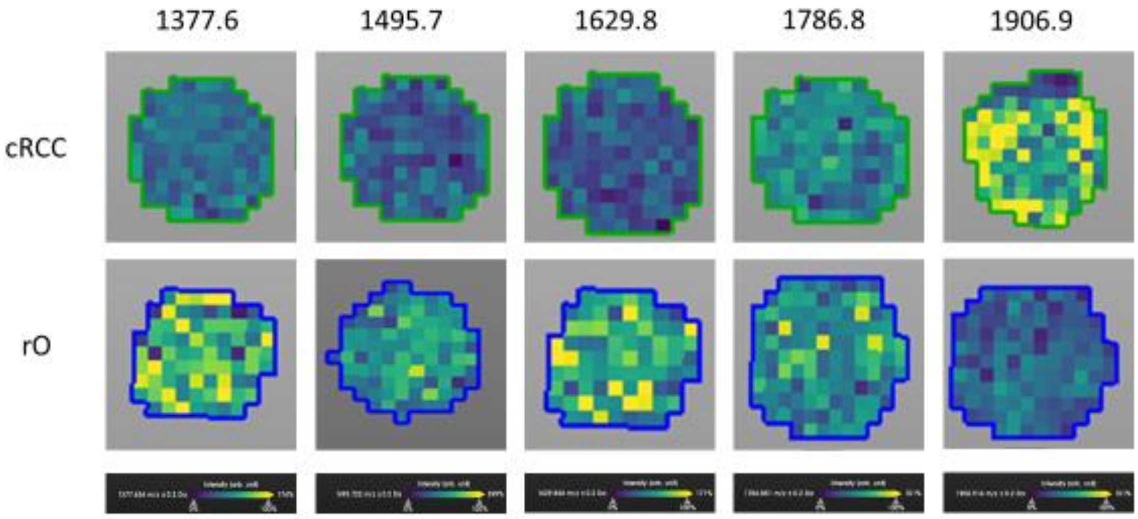

Suppl. Figure 1: Examples of Mass spectrometry Images of selected m/z peaks in cRCC and rO.

The intensity and distribution of the six selected m/z peaks in cRCC (green outline) and rO (blue outline) are displayed. One representative core of the tissue microarray is highlighted.

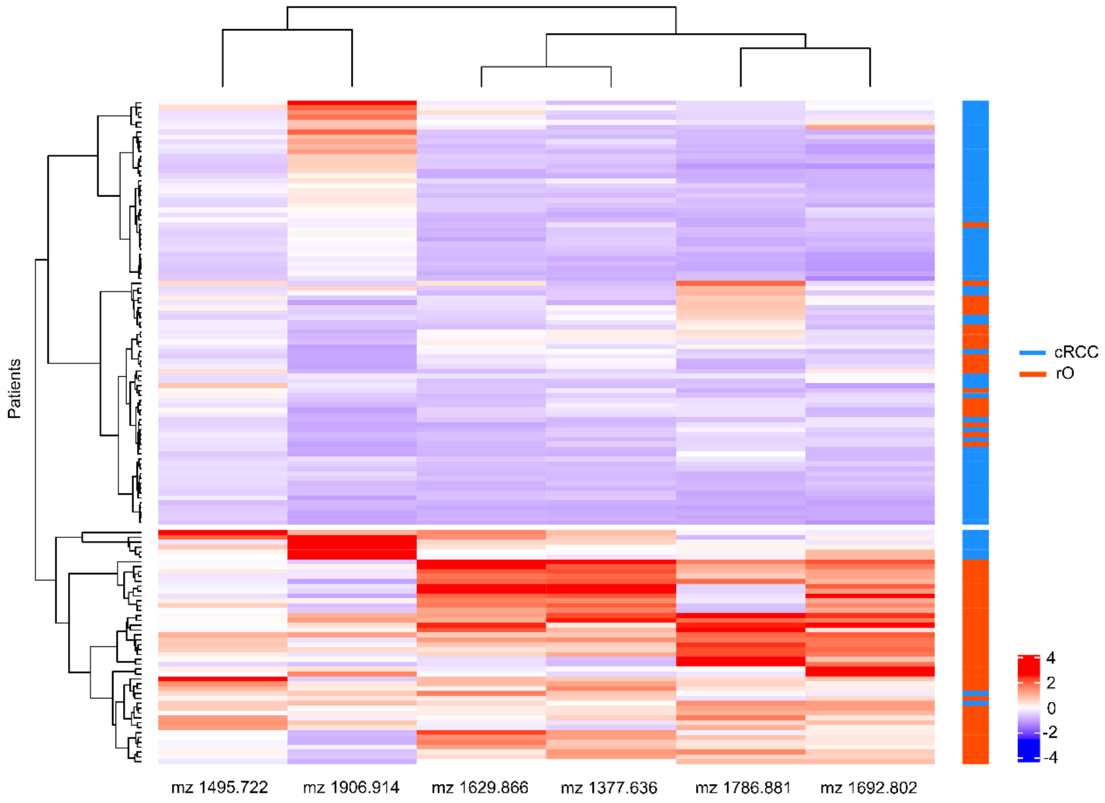

Suppl. Figure 2: Hierarchical unsupervised cluster analysis of selected m/z peaks in cRCC and rO.

Hierarchical unsupervised cluster analysis revealed two main clusters. The supper cluster is enriched with cRCC (blue), while the lower cluster is enriched with rO (red). All but m/z peak 1906.9 showed a higher intensity in rO compared to cRCC.

Suppl. Table 1: Staining properties

Antibody	Company	Clone	Pretreatment	Buffer incubation time (min)	Antibody incubation time (min)	Dilution
CD 117	Dako	c-Kit	Tris/Borat/EDTA, pH 8.4	48	24	1:100
CK 7	Ventana	SP52	Tris/Borat/EDTA, pH 8.4	32	24	RTU
Kaliumhexacyanoferrate	Merck					
Iron(III)-chloride	Merck					
Kernechtrot	Merck					
